# An Educational Intervention to Train Professional Nurses in Promoting Patient Engagement: A Pilot Feasibility Study

**DOI:** 10.3389/fpsyg.2016.02020

**Published:** 2017-01-10

**Authors:** Serena Barello, Guendalina Graffigna, Giuliana Pitacco, Maila Mislej, Maurizio Cortale, Livio Provenzi

**Affiliations:** ^1^Department of Psychology, Università Cattolica del Sacro CuoreMilan, Italy; ^2^Azienda Sanitaria Universitaria Integrata di TriesteTrieste, Italy; ^3^0-3 Center for the at-Risk Infant - Istituto di Ricovero e Cura a Carattere Scientifico (IRCCS) Eugenio MedeaBosisio Parini, Italy

**Keywords:** patient engagement, patient engagement measurement, nursing staff, hospital, professional-patient relation, empowerment, educational program, patient activation

## Abstract

**Introduction:** Growing evidence recognizes that patients who are motivated to take an active role in their care can experience a range of health benefits and reduced healthcare costs. Nurses play a critical role in the effort to make patients fully engaged in their disease management. Trainings devoted to increase nurses' skills and knowledge to assess and promote patient engagement are today a medical education priority. To address this goal, we developed a program of nurse education training in patient engagement strategies (NET-PES). This paper presents pilot feasibility study and preliminary participants outcomes for NET-PES.

**Methods:** This is a pilot feasibility study of a 2-session program on patient engagement designed to improve professional nurses' ability to engage chronic patients in their medical journey; the training mainly focused on passing patient engagement assessment skills to clinicians as a crucial mean to improve care experience. A pre-post pilot evaluation of NET-PES included 46 nurses working with chronic conditions. A course specific competence test has been developed and validated to measure patient engagement skills. The design included self-report questionnaire completed before and after the training for evaluation purposes. Participants met in a large group for didactic presentations and then they were split into small groups in which they used role-play and case discussion to reflect upon the value of patient engagement measurement in relation to difficult cases from own practice.

**Results:** Forty-six nurses participated in the training program. The satisfaction questionnaire showed that the program met the educational objectives and was considered to be useful and relevant by the participants. Results demonstrated changes on clinicians' attitudes and skills in promoting engagement. Moreover, practitioners demonstrated increases on confidence regarding their ability to support their patients' engagement in the care process.

**Conclusions:** Learning programs teaching nurses about patient engagement strategies and assessment measures in clinical practice are key in supporting the realization of patient engagement in healthcare. Training nurses in this area is feasible and accepted and might have an impact on their ability to engage patients in the chronic care journey. Due to the limitation of the research design, further research is needed to assess the effectiveness of such a program and to verify if the benefits envisaged in this pilot are maintained on a long-term perspective and to test results by employing a randomized control study design.

## Introduction

Effectively managing chronic diseases and the burden they put on patients and families, clinicians, and healthcare organizations is currently a major concern for health policy makers (Vogeli et al., 2007). Promoting patient engagement is increasingly acknowledged as a way to address the challenge of chronic conditions (Hibbard and Greene, 2013; Graffigna et al., 2014) and it has been widely advocated as a crucial component of patient-centered models of sustaining healthcare innovation (Thomson et al., 2005; Washington and Lipstein, 2011). Moreover, embracing the patient engagement paradigm is an ethical priority for healthcare systems across countries (Sherman and Hilton, 2014; Solomon et al., 2016; Weil, 2016): it is well-known as a key strategy to include the patients' preferences and expectations in services' design and delivery thus maximizing their clinical effectiveness (World Health Organization, 2002; Eldh et al., 2004; Koloroutis, 2004; Elwyn et al., 2007; Jordan et al., 2008; Eaton et al., 2015; Fisher et al., 2016).

Research has validated this perspective and it has been demonstrated that enhancing patient engagement might increase patients' motivation toward treatments and the care process (Mosen et al., 2007), improve treatment outcomes (Hibbard and Greene, 2013), and generate greater satisfaction for received care (Tobiano et al., 2015). Lastly, engaging patients in their care might contribute to the system's sustainability through a reduction of healthcare services use (Remmers et al., 2009).

### Nursing: a key to patient engagement

In the midst of healthcare transformation and evolution, where patient engagement is an imperative to catalyze the delivery system reform (Carman et al., 2013; Fisher et al., 2016), nursing plays a critical role in the effort to make patients fully engaged in their disease management (Lammon et al., 2010; Barello et al., 2012). Professional nurses across Western countries have a well-established tradition of engaging patients in the medical course and are trained to embrace clinical models which endorse “autonomous and collaborative care of patients of all ages, families, groups, and communities, sick or well and in all clinical settings” (International Council of Nurses, 2010; Pelletier and Stichler, 2013). The concept of relationship-based care constitutes a milestone of the nursing care paradigm with several theories supporting the nurse-patient partnership as the foundation for effective care delivery (Koloroutis, 2004; McCormack and McCance, 2006; Sahlsten et al., 2007; Barello and Graffigna, 2015b). Through the relational and educational aspects of nursing care, nurses have the opportunity to build care relationships that support patient engagement, which ultimately lead to improved patients' quality of life even when life-threatening chronic conditions occur (Gruman et al., 2010; Jerofke et al., 2014; Barello and Graffigna, 2015a; Jenerette and Mayer, 2016). In their clinical encounters, nurses are recognized to be key persons whose strategies might hugely affect the patient's level of engagement in the care process (Jordan et al., 2008; Kutney-Lee et al., 2009; Laschinger et al., 2010; Deyo et al., 2016; Morath and Braaten, 2016). Research on the patients' perspective showed how the patients' perception of professionals' positive attitude toward their self-management behaviors is associated with higher level of patient engagement (Graffigna et al., 2016) and adherence to medical prescriptions (Chan et al., 2009; Schmidt et al., 2012) thus confirming the crucial role of nurses who are often in charge of self-care interventions.

Particularly, scholars (Laschinger et al., 2010; Pelletier and Stichler, 2013) agree that nurses can engage patients by enacting different behaviors and relational strategies: (1) sensitizing patients to be aware of their active role in managing their health and in playing an active part in their treatment plans and decisions; (2) providing patients with trustable information, resources, and support in order to enhance their level of health literacy; (3) stimulating the patients' autonomy in treatment decision making; (4) facilitating exchanges within the formal and informal patients' care network (health providers and family); (5) coordinating care delivered by other providers. For these reasons, nurses are today increasingly valued as professionals qualified to provide personalized and flexible care, which are fundamental for a patient engagement approach to healthcare (Needleman and Hassmiller, 2009; Naylor et al., 2011; Lewis et al., 2016).

### Barriers to patient engagement in the patient-nurse relationship

Despite the growing awareness that engaging patients in their care process should be a nursing priority, considerable barriers related to its effective realization still exist and are reported in literature. For instance, Wellard (2001), Wellard et al. (2003) showed how poor communication between patients and nurses, limited privacy, and task-focused nursing practice might hinder the patients' possibility to engage in effective disease management. According to other scholars (Eldh et al., 2006), patients tend to be disengaged in their care when they do not feel in an equal relationship, when there is a lack of information about their health condition or they experience a paternalistic attitude by their health providers. Previous research also identified patients' dissatisfaction with the limited opportunities given to them to be actual partners in their own healthcare management (Eaton et al., 2015). Probably, this is due to the fact that some health practitioners do have a vision for partnership, but frequently lack the practical resources or pragmatic skills required to actualize this principle in their daily practice (Wellard and Street, 1999; Rasmussen et al., 2001; Wellard and Rushton, 2002; Wellard et al., 2003). Then, although professionals might value and embrace the engagement philosophy, they may experience some tensions between their professional role and the raising patients' desire for autonomy and participation in the care decisions. This may limit professionals in sustaining patient engagement as well as it may be perceived as a potential attack to the established patient-clinician relationship (Kennedy et al., 2007). Moreover, lack of theoretical knowledge might impact nurses' awareness about the need to plan deliberate strategies for effectively engaging patients along their care journey (Sahlsten et al., 2008).

### Collecting patients' experience of engagement: the value of patient engagement measures to support nurses' action

Modern healthcare systems are increasingly recognizing the need for paying attention to the patients' engagement expectations in order to best align services with patients' needs (Elwyn et al., 2007; Black, 2013; Graffigna and Barello, 2016). Patient engagement measures have the potential to drive changes in how the healthcare system is organized and delivered and might contribute to actually realize the “patient engagement promise.” Moreover, patient engagement measurements—initially developed for use in research (Hibbard et al., 2005; Graffigna and Barello, 2016)—have recently started to be considered a mandatory requirement for health professionals to enhance clinical management (Graffigna and Barello, 2016; Hibbard et al., 2016). Adopting this view also implies to consider that patients' engagement expectations might vary depending of the phase of the patients' medical journey (Barello et al., 2015b). Moreover, the patients' conception of what it means to be engaged might change widely, from being welcomed to be an active part of treatment decision and care plans (Thompson, 2007). This means that professionals' skills and actions need to be balanced and attuned with the expertise and expectations of patients. Capturing the patient engagement experience, for this reason, cannot be limited to the professionals' sensitiveness to patients' illness experience; rather, it should be systematically conducted by adopting dedicated assessment tools which are able to collect patient engagement levels. Despite resistances to their adoption still exist, clinicians increasingly recognize the benefits of collecting their patients' engagement expectations. Particularly, the call for patient engagement measures adoption has been sustained by evidences that show they can be of benefit for multiple purposes (Simmons et al., 2014; Graffigna and Barello, 2016; Hibbard et al., 2016; Roberts et al., 2016): (1) a process or outcome measure to determine the patients' characteristics that may predict their level of engagement and their risk for dis-engagement, (2) a tool to personalized interventions based on the individual's ability for self-management (3) an outcome measure for evaluating the performance and effectiveness of healthcare interventions, comparing pre- to post-intervention assessments of one patient's level of engagement; (4) an instrument to optimize and tailor professionals' relational and communicational strategies and, potentially, to improve the effectiveness of their actions.

In the light of these premises, the “measurement act” can become a precious occasion for nurses to feel empowered in their clinical effectiveness and to have concrete guidelines about how to achieve positive relationship with their patients and provide more patient-centered care. However, the innovation potentials offered by the adoption of patient engagement measurements can only be realized if clinicians can understand the values of these data in patient care and start collecting and utilizing this information in their daily practice. Indeed, a key issue limiting successful implementation of patient engagement measures is the clinician's lack of knowledge and awareness about how to effectively utilize these data in the clinical management of their patients. All these factors appear to stress the need to train professional nurses in introducing and benefit from patient engagement measures in their daily clinical practice.

### The present study

In order to enhance professional nurses' ability to make their patients effectively engaged in their medical course, the authors (GG and SB) developed a novel program designed to train nurses in patient engagement strategies and to effectively adopt patient engagement measures in their routine practice (NET-PES program). From a theoretical point of view, the implementation of patient engagement measurement in routine patient care represents a significant change to clinical practices of both individual clinicians and healthcare organizations. Changing the organizational culture of healthcare systems is well-known to be a challenge. The phases of the change process, namely dissemination, adoption, implementation, and continuation (Fleuren et al., 2004), can be applied specifically to the process of implementing patient engagement measures in clinical practice, in the light of specific organizational and clinical issues. The training of clinicians represents the first step of dissemination.

The NET-PES program is based on the assumption that clinicians who are sensitized to the value of patient engagement, who have adequate theoretical and practical knowledge about this concept, who are provided with communication and relational skills and have reasonable confidence in their own abilities to engage patients are more likely to correctly assess and thereby to better respond to patients' engagement needs and expectations. Moreover, a central asset of the course is that without providing professionals with reliable measures for capturing their patient engagement level, they cannot succeed in tailoring their own actions on patients' care expectations and preferences. The course was developed according to extensive research about patient engagement and the factors enabling it and designed to be highly participative and interactive among participants (Hibbard et al., 2005; Graffigna and Barello, 2015a). This is a report of feasibility testing of the program along with examining potential impact and participant satisfaction.

## Materials and methods

We conducted a pre-post pilot study evaluating the feasibility of the NET-PES program. Self-report questionnaires were completed before and after the workshop for evaluation purposes. To guarantee data confidentiality and assure anonymity, each participant was assigned with a random identification number in order to track data from pre- and post-questionnaires. All nurses working with chronic patients at the Azienda Sanitaria Universitaria Integrata di Trieste (Italy) were contacted and recruited through e-mail invitations sent to the hospital units, to service medical directors and to nurse managers and they were invited to participate in the course. Only nurses having work experience with chronic patients were considered eligible for the training program. Eligible nurses interested in learning more were invited to an information session. Recruitment materials emphasized the voluntary nature of the program.

Due to the fact that it is an observational design with education as the intervention it didn't require any ethical approval by the hospital institutional review board according to the institutional and national requirements. However, we collected by all participants written informed consent for confidentiality issues. We confirm that all ongoing and related trials for this observational educational intervention will be submitted to Ethical approval and registered in ClinicalTrials.gov.

### The NET-PES program description

According to the principles of patient engagement and activation theories (Hibbard et al., 2005; Graffigna and Barello, 2015a), a theoretically-driven educational training for nurses working with chronic patients entitled “Nurses Engagement Training in Patient Engagement Strategies” (NET-PES) was designed, delivered, and tested by researchers who have experience in adult training programs and patient engagement theories. Moreover, specific inputs were given from clinicians (GP, MM, MC, LP), which contributed to outline the main educational needs of the target participants, reviewed didactic materials and training scenarios. To guarantee adequate participation, a maximum number of 25 participants was set for each edition of the workshop. Teaching methods included adult teaching learning principles (Colliver, 2000) and provided an experiential learning environment where nurses were encouraged to share their professional experience and reflect on patient engagement strategies they enact in their daily clinical practice.

The program content was articulated into four main themes: (1) *setting the scene*: a didactic overview covering the core principles of patient engagement and activation theories and the evidence base for improving nurses' knowledge and skills to engage patients in their care; (2) *the whys for adopting patient engagement measures*: an outline of the main evidence supporting the value of patient engagement measures and the ben'efits of using them in clinical practice; (3) *acquiring practical skills to use patient engagement measures in clinical practice*: familiarization with the measures, understanding of clinically important differences among the patient engagement profiles, how to adapt communication and relational style to different levels of engagement, and developing shared strategies to guide potential nurses' actions based on patients' scores; (4) *from theory to practice*: overview of the most effective patient engagement interventions currently available in the scientific literature and tools for guiding clinical actions aimed at promoting patient engagement. Table 1 describes in details the contents, the learning objectives and the main educational techniques featuring the program.

**Table 1:** **Program sessions' description**.

**Sessions**	**Content**	**Learning objectives**	**Learning activities**
Day 1	**Setting the scene** A didactic overview covering the core principles of patient engagement and activation theories and the evidence base for improving nurses' knowledge and skills to engage patients in their care	Enhance nurses' motivation and sensitiveness toward the value of engaging patients in the care process Introduce the theoretical underpinnings at the base of the patient engagement paradigm Make nurses aware of their crucial role in realizing patient engagement and catalyzing organizational change	Didactic theoretical presentations, group discussion, narratives Role-playing, clinical case discussions, group and facilitators feedbacks, wrap-up
	**The whys for adopting patient engagement measures** An outline of the evidences supporting the value of patient engagement measures (PAM and PHE-s)	Provide nurses with the evidence base for adopting patient engagement measures in the clinical practice Discuss the clinical and relational value of including patient engagement measures in the routine patients' assessment along with other clinical parameters	
Day 2	**Acquiring practical skills to use patient engagement measures in clinical practice** Experience-based session dedicated to make nurses experience the use of patient engagement measures and pragmatically reflect upon their use in the relationship with patients	Familiarize nurses with the main patient engagement measures and make them experience how to use data obtained through them Teach nurses how to use patient-reported outcomes as a relational instrument within the interaction with patients Promote nurses' competences in patient centered communication and relational skills basing on the patient engagement profiles Sensitize nurses to align their communication and relational style to the level of patient engagement	Didactic theoretical presentations, standardized patient exercises, vignettes, clinical case discussions
	**From theory to practice** Description of the main interventions currently available for promoting patient engagement of provision of a toolbox for orienting clinical practice aimed at sustaining patient engagement	Provide nurses with validated and evidence-based intervention strategies grounded in the literature of patient activation and engagement Let nurses experience tools and exercise with simulated patients	Didactic theoretical presentations, role-playings, wrap-up

Didactic presentations were constantly alternated with interactive discussions of clinical cases of real patients with different clinical conditions. The cases included a summary of the medical history, patient engagement profiles' card, and linkage of the patient engagement profiles to patients' clinical parameters. These allowed reflecting upon the value of collecting patient engagement measures to address the patients' needs across patient engagement profiles and, consequently, to tailor the interventions. Moreover, simulated clinical consultations enacted by a patient-actor followed by debriefing with facilitators allowed participants to share knowledge, to receive live peer feedback and to experience a collaborative learning context with colleagues working in different clinical units. The interactive fashion of the program was encouraged by facilitators and provided rich contents for the course.

The supporting materials used in the program included: the main measures of patient engagement currently available in the scientific literature (i.e., Patient Activation Measure by Hibbard et al., 2005; Patient Health Engagement Scale by Graffigna et al., 2015b), the case studies discussed and memory-aid materials including the scales' scoring system, guidance on changes in patients' needs across the patient engagement levels, and related suggested actions, and scientific articles discussing the evidence base that oriented the program's contents.

### Participants' outcomes and program evaluations

Feasibility was evaluated including process measures (such as participants' attendance, completion rates of assessment measures, and satisfaction with the program). Nurses engagement attitudes and skills were assessed using self-report measures administered before (T_0_) and at the end of the program (T_1_). The second day of the course was delivered 1 month after the first session. Socio-demographic and professional characteristics (i.e., age, gender, discipline, year of professional experience,) were collected before the delivery of the program.

Pre- and post-evaluation surveys included the following measures:

- The 13-item version of the *Clinician Support Patient Activation Measure (CS-PAM)* (Rademakers et al., 2015), which assesses clinicians' believes and attitudes toward the importance of patient self-management behaviors and his/her active role in the care process. The items of the CS-PAM are aimed at assessing the degree to which practitioners value patient activation behaviors as important (Hibbard et al., 2010). Particularly CS-PAM items might be grouped into four categories of increasing level of patient's activation behaviors that clinicians might endorse (items 1, 4, 5, 8: “*patient should have the knowledge and behave in order to prevent or minimize symptoms associated with their health condition*” (adherence); items 2, 6, 7: “*patient can make independent judgment and action*” (independency); items 9, 10, 11: “*patient is able to take an active role during the consultation*” (partnership); items 3, 13: “*patient should be an independent information seeker*” (information seeker). Each item is scored using a 5-point Likert scale (1, Strongly disagree; 2, Disagree; 3, Agree; 4, Strongly agree; 5, Not applicable).
- *Clinicians Competence in Patient Engagement Strategies (CC-PES)* is an ad-hoc scale developed for the aims of the program that scores clinicians on 9 items, which equate to their perceived confidence in enacting 9 key strategies to promote patient engagement. Each item is scored using a 5-point Likert scale (1, Not at all confident; 2, Not very confident; 3, Neutral; 4, Confident; 5, Very confident). Competences assessed are the followings: (1) *assess the level of patients' engagement*; (2) *generally support patient engagement*; (3) *motivate patients in following medical prescriptions*; (4) *inform patients about disease and treatments*; (5) *assess patient's health literacy*; (6) *empathize with patients*; (7) *assess and manage difficult patients' emotions*; (8) *effectively communicate with patients and their families*; (9) *effectively relate with patients and their families*.
- *Participants' experience and satisfaction questionnaire*: the post-questionnaire was created to obtain rating and qualitative reports for participants on their experience and satisfaction with the NET-PES program and recommendations for future improvements. Particularly the survey consisted of six items, including six questions rated with a 10-point Likert scale assessing nurses' satisfaction with the course in term of general satisfaction, interest, usefulness, quality of teaching, quality of didactic materials, and quality of practical sessions. Open-ended questions were also used to gather information on what participants liked most and least about the program (in terms of both didactic contents and teaching methods) and to obtain specific recommendations to improve the program itself.

### Fidelity assessment

The research team monitored the program fidelity by implementing monthly supervision and protocol review sessions including the principal investigators, co-investigators, and program facilitators. In addition, the principal investigator attended at least one session of the course and provided feedbacks to program facilitators. Ongoing review of the program sessions through the study period revealed both the strengths and weakness of the protocol design and intervention delivery. This pilot feasibility work has been intended to form the basis for subsequently modifying the design of the NET-PES program for a larger clinical trial.

### Data analysis

Participants' socio-demographic and professional features and satisfaction items were analyzed through descriptive statistics. To determine the internal consistency of the adopted measures, Cronbach's α was used. As the CC-PES was ad-hoc developed for the purposes of the present study, the factorial structure was assessed with a principal component analysis (PCA). Paired-sample *t*-tests were used to compare T_0_ and T_1_ mean scores of CS-PAM and CC-PES. Moreover, two separate multivariate analyses of variance (MANOVAs) were used to assess pre-to-post changes in CS-PAM scales and CC-PES items. To adjust for multiple testing, consistent with recent literature (Provenzi et al., 2015), the Benjamini-Hochberg false discovery rate (FDR) procedure was used, setting *q* < 0.05. Basic assumptions for the use of MANOVA were checked, including assessment of normal distribution of variables through the Kolmogorov-Smirnov test. The Statistical Package for Social Sciences (SPSS) version 21.0 for Windows was used for data analysis. Open-ended responses were transcribed and analyzed using a content analysis approach. Two researchers (SB and GG) independently read and coded the open responses in order to detect recurring themes representative of the participants' viewpoints and recommendations for future improvements.

## Results

### Preliminary results

In the following paragraphs, data collected from two subsequent editions of the program are presented.

#### Participants' characteristics

Overall, 49 nurses participated in the educational program. Among them, 46 completed both pre- and post-questionnaires and attended both sessions of the program. Of the three missing questionnaires, three pre-questionnaires were not filled because participants arrived late. Participants with missing questionnaires did not differ from included subjects for socio-demographic and professional characteristics. Mean age of included professionals was 47 years (*SD* = 7.08, range 25–58). Mean years of professional experience was 23.9 (*SD* = 8.64, range 3–35). The majority of participants were female (78.3%).

#### Instruments reliability

The CS-PAM pre- and post-training Cronbach's alpha was 0.89 and 0.85, respectively. Cronbach's alpha for the CC-PES was 0.87 (T_0_) and 0.89 (T_1_). As such, reliability was high for both instruments. The PCA revealed a one-factor structure for the CC-PES, with PC1 explaining 51.2% of variance (eigenvalue = 4.61).

### Program outcomes

The mean scores of CS-PAM, *t*_(45)_ = 4.05, *p* = < 0.001, and CC-PES, *t*_(45)_ = 3.48, *p* = 0.001, increased from T_0_ to T_1_.

#### CS-PAM outcomes

The attitudes of participants toward patient activation and self-management behaviors changed from the beginning to the end of the program, *F*_(4, 42)_ = 5.08, *p* = 0.002, $\eta_{p}^{2}$ = 0.33. Scores improved for each of the CS-PAM categories (Table 2). Testing for False Discovery Rate (FDR) did not affect the results and no significant mean comparison was excluded.

**Table 2:** **Descriptives for CS-PAM scales and mean comparison statistics**.

**Scales**	**T**_**0**_				**T**_**1**_				**Mean comparisons**	
	**Min**	**Max**	**Mean**	***SD***	**Min**	**Max**	**Mean**	***SD***	***F***	***p***
Adherence	2.75	4.00	3.58	0.43	2.50	4.00	3.76	0.37	7.72	0.008
Independency	2.00	4.00	3.38	0.51	2.67	4.00	3.72	0.36	17.29	0.000
Partnership	1.75	4.00	3.49	0.55	2.75	4.00	3.73	0.38	8.58	0.005
Information seeker	1.00	4.00	3.05	0.62	2.50	4.00	3.38	0.38	11.83	0.001

#### CC-PES outcomes

Participants also reported improvements in their confidence toward enacting patient engagement strategies, *F*_(9, 37)_ = 2.26, *p* = 0.039, $\eta_{p}^{2}$ = 0.36. Specifically, an increase was observed in 8-out-of-9 skills depicted by the CC-PES (see Table 3). Testing for FDR did not affect the results and no significant mean comparison was excluded.

**Table 3:** **Descriptives for CC-PES items and mean comparison statistics**.

**Items**	**T**_**0**_				**T**_**1**_				**Mean comparisons**	
	**Min**	**Max**	**Mean**	***SD***	**Min**	**Max**	**Mean**	***SD***	***F***	***p***
Assessing the level of patient engagement	3.00	5.00	3.76	0.52	3.00	5.00	4.09	0.66	7.31	0.010
Promoting patient engagement	2.00	5.00	3.78	0.63	3.00	5.00	3.96	0.59	3.04	0.090
Motivating patients in following medical prescriptions	2.00	5.00	3.65	0.71	3.00	5.00	4.02	0.54	10.58	0.002
Informing patients about disease and treatments	2.00	5.00	3.80	0.62	3.00	5.00	4.09	0.51	5.64	0.022
Assessing patient's health literacy	2.00	5.00	3.72	0.72	3.00	5.00	4.02	0.54	6.04	0.018
Empathizing with patients	2.00	5.00	3.93	0.68	3.00	5.00	4.24	0.60	8.08	0.007
Assessing and managing difficult patients' emotions	2.00	5.00	3.50	0.75	3.00	5.00	3.80	0.65	6.04	0.018
Effectively communicating with patients and families	2.00	5.00	3.80	0.69	3.00	5.00	4.09	0.55	4.68	0.036
Developing/maintaining relationship with patients and families	3.00	5.00	4.15	0.56	3.00	5.00	4.37	0.53	4.48	0.040

### Participants' experiences and satisfaction

Overall, on a scale from 1 to 10, satisfaction with the program was high (mean = 8.62), as well as the level of participants' interest toward the course's contents (mean = 8.76), the perceived course usefulness (mean = 8.65), the quality of teaching (mean = 9.07), the quality of the didactic materials (mean = 8.77) and of the interactive sessions (mean = 8.79).

Moreover, from the qualitative analysis of participants' responses, several themes emerged to describe the most useful aspects of the program. Participants gave highly positive feedback after the program. Participants found the course to be interesting, useful, enjoyable, informative, and highly relevant to their own nursing practice and for teaching junior staff.

Summarizing and analyzing participants' written reports resulted in the following achievements:

*Learning through sharing*. Particularly, nurses reported highly positive feelings related to the opportunity to *share and discuss their experience* with colleagues belonging to different clinical units within a safe and stimulating learning environment. The heterogeneous group nature of the program was, in this sense, particularly valued as a fundamental factor to promote a safe climate of discussion and feedback.
*Learning by doing*. Moreover, participants highly valued the possibility to participate in role-playing and case scenarios with simulated patients. This allowed them to directly experience contents explained during the didactic sessions and to reflect upon them by receiving feedbacks from other participants and facilitators.
*Valuing the nursing professionalism*. The *valorization of the nursing profession* and the capitalization on the past professional experience was then considered a core aspect of the program and was highly appreciated by participants who felt professionally recognized. The contents of the course were perceived as a systematization and valorization of the daily work of nurses.
*Time for self-reflection*. Furthermore, the possibility to reflect while learning allowed nurses *to question and discuss daily practices with patients and clinical hidden assumptions*. Particularly, participants reported as a value of the program the space given to reflect upon clinical models and cultures that often are enacted without any critical thinking. Nurses commented on the importance to build a culture of patient engagement, which emphasize patients' autonomy, and is respectful of the patients' needs along the care process.
*Innovation for the nursing paradigm*. Finally, the course was also perceived useful due to the *innovativeness* of its contents. This was perceived to be a step forward from the epistemology of patient-centeredness to concrete strategies of action to actually promote the centrality of people along the care process. The possibility to experience and understand the *relational value* of patient engagement measures was perceived as a useful aspect of the training. Having assessment measures that concretely helps clinicians in detecting the patients' needs and expectation of being involved in their care allowed clinicians to reflect upon their relational strategies to really address the needs of patients.
*Suggestions for improvements*. Regarding the least favorite aspects of the program and recommendation for the future, participants least liked its brief duration, and reported to desire more individual feedback on their patient engagement during the course and in their implementation in the actual clinical practice. Finally, participants recommended that the program in the future include the testimony of nurses who successfully employed patient engagement strategies in the clinical work with chronic patients and additional guidance on communication and relational strategies to improve the engagement of both patients and their caregivers.

Selected quotes and overall descriptive themes from open-ended questions exploring participant satisfaction and recommendation for future improvements are listed in Table 4.

**Table 4:** **Selected quotes and overall descriptive themes representing nurses satisfaction with the program and recommendation for future improvements**.

**Domains**	**Themes**	**Sample quotes**
Favorite aspects	Learning through sharing	*Among the most useful aspect of this course I identify the group as a fundamental source of exchange and feedback (nurse, male, 27 years of professional experience).* *This is a different—but really effective—way to learn something new (nurse, female, 15 years of professional experience).* *The group allowed us to share our daily practice, by valuing each one experience (nurse, female, 25 years of professional experience)*
	Learning by doing	*I really appreciated the experiential moment. It was useful to observe encounters between a nurse and a patient from outside (nurse, female, 8 years of professional experience).* *It was really useful to experience a simulated consultation with a patient. It allows me to actually put in the patient shoes (nurse, female, 29 years of professional experience)*
	Valuing the nursing professionalism	*I appreciated that in this course emerged a strong valorization of the role of nurses…(nurse, female, 17 years of professional experience).* *This course helped me in systematizing what we (the nurses) just do when we have to motivate and engage patients in the medical course (nurse, male, 13 years of professional experience).* *I felt recognized as a key player in the care team (nurse, female, 4 years of professional experience)*
	Time for self-reflection	*I learned the importance of taking time to reflect upon our own clinical practice (nurse, male, 30 years of professional experience).* *I took home a great awareness about the complexity of the patient inner world. This means that what we (i.e., nurses) perceive is not necessarily aligned with the real patient experience (nurse, female, 19 years of professional experience)*
	Innovation for the nursing paradigm	*I did not expect that this course could offer concrete tools for the nursing practice (nurse, female, 22 years of professional experience).* *We are used to learn theories about putting the patient at the center of their care…it is much rarer to discuss and experience concrete strategies (nurse, male, 8 years of professional experience).* *I did not expect that this course could offer concrete tools for the clinical practice; I was not used to conceive patients' assessment as a relational instrument (nurse, female, 14 years of professional experience).* *I understood that patients' assessment is not a mere bureaucratic act…it could be useful in the clinical daily practice (nurse, female, 34 years of professional experience)*
Least favorite aspects	Brief duration Not enough personal feedback	*I would have preferred this program was longer (than two sessions). I wanted to talk more on each subject (nurse, female, 22 years of professional experience).* *I was never sure if the role-playings we enacted in the sessions was right (nurse, male, 19 years of professional experience).* *I would like to share my actual clinical experience to see how much I have improved my skills after the course (nurse, male, 26 years of professional experience)*
Recommendations for future improvements	Peer testimony	*To listen to successful implementation experience of others (nurses) who employed in their practice the acquired patient engagement skills. I would find their voice inspiring (nurse, female, 12 years of professional experience)*

## Discussion

In this paper we described the preliminary examination of the feasibility of a 2-session program (NET-PES) designed to train nurses in adopting patient engagement measures to enact patient engagement strategies. Healthcare quality improvement efforts are increasingly focused on chronic illness care, and there will undoubtedly be heightened interest in monitoring the clinicians' performance in supporting patient engagement in the management of their health condition; at the same time, healthcare organizations need to train their clinicians in order to provide them knowledge and skills to effectively support the patient active role. Within this context, nurses are crucial actors for sustaining this paradigmatic revolution and promoting the imperative of patient engagement. Training the next generation of clinicians—and of nurses as one of the main patient's navigators along the complexities of the current healthcare environment—means not only to emphasize the relevance of supporting patients in self-care and self-management skills, but also to sustain the acquisition of skills and strategies to monitor and encourage patient engagement along the medical course.

This pilot feasibility study demonstrated that NET-PES was associated with observable improvements in nurses' attitudes and skills for promoting patients' engagement in chronic care management. Also, qualitative feedback from participants revealed that the program made them more confident in delivering patient engagement actions. These findings, despite the small sample size, suggest preliminary evidence that a brief educational intervention designed to train nurses in patient engagement assessment and improvement is not only feasible but holds promise toward potentially improve clinicians' patient engagement strategies. The achieved results also highlighted the potential for the NET-PES program to contribute to efforts aimed at improving the quality of healthcare for persons with chronic conditions. To date, interventions in the area of patient engagement have largely concentrated on improving health providers' communication and relational behaviors and their empathy in the delivery of care (Kennedy et al., 2005; Morriss et al., 2006; Levinson et al., 2010; Otero-Sabogal et al., 2010; Cunico et al., 2012; Légaré et al., 2012). NET-PES was aimed to complement these established efforts by focusing on training engagement assessment measures particularly useful to identify the patients at high risk of disengagement ant to adapt the health professionals' relational strategies of the basis of the patients' priorities and engagement needs.

Consistent with prior studies about interventions aimed at educating providers on supporting patient activation and engagement (Greene et al., 2007; Lamiani et al., 2012; Barello et al., 2015a), our findings may indirectly suggest that this program might be potentially helpful to enhance patients' healthcare outcomes. From this perspective, previous studies have demonstrated a positive correlation between the patients' perception of their clinicians' support for autonomy in self-care and the patients' level of engagement in self-management behaviors and adherence to treatments (Graffigna et al., 2016). There are also studies that discussed the role of health practitioners' beliefs about the importance of patients being active agents in managing their health condition (Greene and Yedidia, 2005; Blakeman et al., 2006). The encouraging results of this study, unless preliminary, support the value of designing, and implementing educational initiatives dedicated to nurses among the actions to realize the patient engagement imperatives. In line with other experiences across countries, nurses may be envisaged as a powerful resource to promote organizational care models where this professional figure can absolve and foster the implementation of integrative approach to patient's care.

Despite the promising nature of these results, there are a number of limitations that warrant consideration. First, the use of pre-post design prohibits any inference of program effectiveness due to the lack of a comparison group and due the challenges to internal validity of this king of research design as suggested by Vockell and Asher (1995). Moreover the single site study limits results' generalizability. This study design—frequently adopted in research testing the feasibility and potential impact of educational interventions (Hulsman et al., 1999; Berger et al., 2010; Lamiani et al., 2011; Meyer et al., 2011; Bartels et al., 2013; Ledford et al., 2014; Arnold et al., 2015; Shah et al., 2015; Robinson et al., 2016; Viau et al., 2016) is appropriate for the demonstration of feasibility and for initial proof of concept, it is cost effective, and it is pragmatic because it allows for “real world” variability in variables. Consistent with the intent of a pilot study, our goal was to examine feasibility and potential impact. A determination of effectiveness will require a larger an appropriately selected sample, ideally employing a randomized controlled trial with follow-up measures to estimate the maintenance of achieved results across time. Finally, participants in our pilot study were recruited using a convenience sample. As attendees were largely self-selected, the surveyed group might be more open to reflection and self-improvement than a randomly selected group. Moreover, also the Hawthorne effect could have had an impact on the nurses' engagement behaviors. Hence, we are unable to generalize our findings to a different population of nurses. Also the clinicians' self-rating of self-efficacy and attitudes can be considered as a methodological weakness because of the risk of response bias. The nurses involved were not blinded to the intervention and the increase in their self-ratings can therefore reflect a willingness of the responders to show that the training course had been useful. Moreover, it should be noted that nurses' T_0_ scores were high in both CS-PAM and CC-PE (mean pre-training score = 3.43, *SD* = 0.45; mean post-training score = 3.68, *SD* = 0.31): this could be related to a self-selection bias because of voluntary participation and the risk of an over-representation of participants who believed in and supported patient engagement in care should not be completely neglected. Also, the majority of participants were women, and it is possible that men and women may respond differently to the program's stimuli: previous research on patient-centeredness has indeed demonstrated that gender may affect the attitudes of clinicians toward being more or less open to involve patients in the care process (Roter and Hall, 2004; Sandhu et al., 2009). More inquiry is needed to determine how nurses implement teaching principles in their practice and how patient-related factors may affect that implementation.

## Conclusions

These findings provide preliminary evidences that a brief educational program designed to improve nurses' competences in promoting patient engagement is not only feasible, but holds promise toward potentially implementing this training in nursing educational curriculum. If proven in a future empirical trial, NET-PES program might provide a useful contribution to translate the principle of patient engagement from theories into actual practice.

Today's care models require nurses to play many different roles: these include providing high quality and compassionate nursing care; the ability to adopt a multidisciplinary perspective to the patients' care; partnering with patients and their families (Barello et al., 2014, 2015a); valuing the patients' perspective by collecting their care experience in an ecological way (Graffigna et al., 2011, 2015a). For these reasons, embracing a patient engagement care model and the necessary skills to enact it become vital components for the nurses' education. However, to effectively realize these goals, clinicians have to be provided with reliable measures to assess their patient engagement levels and trained to effectively use them. There is an increasing need to provide more resources for effective training in this area if patient engagement is to improve. Such initiatives are also essential to ensure the professional well-being of nurses themselves by valuing and recognizing their crucial role in addressing the challenges of the current healthcare environment. If demonstrated effective, learning programs teaching clinicians how to use and act on patient engagement measures in clinical practice might be a key steps in supporting the realization of patient engagement in healthcare (Barello et al., 2015a; Graffigna et al., 2016). Researchers and clinicians from different clinical areas should collaborate to share good practices and develop guidelines to advance the field of patient engagement and shift from theory into practice.

The concrete implementation of patient engagement interventions and the adoption of patient engagement measures is a dynamic and challenging process, in which different factors play a role at both individual (professional, patients, caregivers) and organizational levels. We focused here on nurse's training, but it is necessary to acknowledge the importance of other factors. First, patients and caregivers' trainings in gaining an active role in their care are of paramount importance for patient engagement successful implementation. Studies exploring patients and caregivers' attitudes clearly state that if their clinicians are open to patient's autonomy and self-care behaviors, this will encourage patients' active involvement in care (Graffigna and Barello, 2015b). Second, an organizational revision of care models (Bosio et al., 2012) and a pragmatic support for collecting and integrating patient engagement measurement into patient records and in the clinical workflow is a required step. Finally, using opinion leaders and specific organizational change strategies also appear pivotal to maximize the possibilities of innovation's adoption.

## Author contributions

GG and SB developed the research design, coordinated data collection and wrote the manuscript. LP performed statistical analyses and give clinical supervision about the contents. GP, MM, and MC revising the work critically for important intellectual content. The authors have approved the article and agree to be accountable for all aspects of the work.

### Conflict of interest statement

The authors declare that the research was conducted in the absence of any commercial or financial relationships that could be construed as a potential conflict of interest.
